# The impact of sleep deprivation on symptoms of anxiety, depression, stress and on the quality of life in medical staff

**DOI:** 10.1192/j.eurpsy.2024.1607

**Published:** 2024-08-27

**Authors:** M. Pavllo

**Affiliations:** ^1^Psychiatric Department, University Center Hospital “Mother Tereza”; ^2^Psychiatric Department, Regional Hospital of Lezhae, Tirana, Albania

## Abstract

**Introduction:**

Sleep deprivation is studied in medical staff, as it is a target group more exposed to chronic lack of sleep compared to the normal population.Chronic sleep deprivation has an important impact in the lifestyle of health workers and in their productivity

**Objectives:**

The study aims to examine the impact of sleep deprivation on medical staff, who work night-shifts and / or 24 hours on the symptoms of anxiety, depression, stress and quality of life.

**Methods:**

This is a quantitative, cross-sectional study. The research instruments used are two: the DASS-42 questionnaire for measuring the level of symptoms of anxiety, depression, stress and the quality of life questionnaire (WHOQOL-Bref ), which was validated before the study. In the study sample participated N = 199 medical staff (primary doctor, resident, nurse) from several specialties. Inclusive criteria are: medical staff, who work night-shifts and / or 24 hours; age 23 - 67 years; have not been previously diagnosed with anxiety disorder and episodes of depressive disorder.

**Results:**

Refering to the DASS-42 scoring, the symptoms of depression in the medical staff are: normal 64.8%, mild 8.5%, moderate 21.1%, severe 4.5% and extremely severe 1%. Refering to the points collected from the DASS-42 questions on the symptoms of anxiety in medical staff, it results: normal 53.3%, mild 8%, moderate 17.1%, severe 14.1% and extremely severe 7.5%. Based on the points collected from the DASS-42 questions on stress symptoms in medical staff, it results: normal 54.3%, mild 18.6%, moderate 17.1%, severe 9% and extremely severe 1%. Also, the lower the level of stress, anxiety and depression the higher the quality of life. (p.01, p.05). Total WHO- Quality of life (F = 3.447, p≤.05) and physical health (F = 6.482, p≤.05) show significant differences between the educational level, where it is higher among medical staff with postgraduate education.

**Conclusions:**

Working night-shifts and/or 24 hours affects the mild and moderate onset of symptoms of anxiety, depression and stress. The level of stress symptoms is perceived higher in females. The overall quality of life is perceived as average according to the Likert scale. Sleep deprivation affects free time. Medical staff have a restricted free time.The level of anxiety, depression and has a direct impact in the quality of life. The overall quality of life and physical health are rated higher in medical staff with postgraduate education.

**Disclosure of Interest:**

None Declared

